# Do UK Allied Health Professionals (AHPs) have sufficient guidelines and training to provide telehealth patient consultations?

**DOI:** 10.1186/s12960-022-00778-1

**Published:** 2022-12-05

**Authors:** Enza Leone, Nicola Eddison, Aoife Healy, Carolyn Royse, Nachiappan Chockalingam

**Affiliations:** 1 Centre for Biomechanics and Rehabilitation Technologies, Staffordshire University, Leek Road, Stoke-on-Trent, ST4 2DF United Kingdom; 2 Royal Wolverhampton NHS Trust, Wolverhampton, WV10 0QP United Kingdom; 3 Dorset County Hospital NHS Foundation Trust, Dorchester, Dorset, DT1 2JY United Kingdom

**Keywords:** Telehealth, Allied Health Professionals, Guidelines

## Abstract

**Objectives:**

The COVID-19 pandemic caused a rapid shift to remote consultations. United Kingdom (UK) NHS Allied Health Professional (AHP) services may have been unprepared for telehealth implementation. This study explored these services’ organisational readiness regarding telehealth guidelines implementation and staff training.

**Methods:**

A cross-sectional online survey exploring available telehealth guidelines and staff training was distributed among UK AHPs and AHP service managers between May and June 2021.

**Results:**

658 participants answered the survey (119 managers and 539 clinicians). Most services, in which telehealth was in place, had implemented telehealth guidelines (clinicians, 64%; managers, 82%), with most guidelines produced by the NHS staff who use them for their consultations. Most clinicians reported that guidelines had ambiguous areas (e.g., regarding protection from litigation and dealing with emergencies), whereas most managers reported the opposite opinion. Guidelines most frequently reported on appropriate telehealth technology and environment for staff and patients, while recommended consultation length and how to conduct telehealth with certain population groups were least reported. Clinicians lacked training in most telehealth aspects, while managers reported that staff training focused on telehealth software and hardware. For both clinicians and managers, training is needed on how to deal with emergencies during telehealth.

**Conclusions:**

UK NHS AHP services are not fully equipped with clear and comprehensive guidelines and the skills to deliver telehealth. Vulnerable people are excluded from current guidelines, which may widen health inequalities and hinder the success of the NHS digital transformation. The absence of national guidelines highlights the need for uniform AHP telehealth guidelines.

**Supplementary Information:**

The online version contains supplementary material available at 10.1186/s12960-022-00778-1.

## Key messages

- Nationally accepted guidelines for AHP telehealth consultations are needed, with collaboration required between the NHS and AHP professional bodies.
- Future telehealth guidelines need to include advice and clear recommendations on patient eligibility criteria, consultation length, training requirements and safety considerations to limit unintended risks to patients, avoid AHPs’ liability exposure and widening health inequalities of vulnerable groups.
- Telehealth implementation processes should be given equal consideration as technological aspects within guidelines and staff training to ensure a successful digital transformation of the NHS AHP services.
- Digital inclusion approaches are needed to ensure that vulnerable population groups at risk of exclusion can benefit from digital technologies as digitally enabled care goes mainstream across the NHS.
- Training should include not only technical but also practical skills in telehealth consultations. AHPs must be trained to respond to emergencies to reduce the risk of unnecessary harm to patients and to improve their confidence and engagement in the usage of telehealth.
- Telehealth education should be incorporated into AHP curricula to equip the future AHP workforce with the knowledge and skills necessary to deliver telehealth.

**Supplementary Information:**

The online version contains supplementary material available at 10.1186/s12960-022-00778-1.

## Introduction

The COVID-19 pandemic has caused a rapid and sudden transition from face-to-face appointments to technology-based patient consultations, known as telehealth [1], in healthcare systems worldwide [2], including the UK National Health Service (NHS). With the need to reduce the infection risk and ensure continuity of care [3], UK healthcare services quickly adapted to scaling telehealth across different services, including Allied Health Professional (AHP) services. This required a massive effort from AHPs, who have over four million patient contacts per week, and resulted in a significant telehealth expansion [4].

The NHS had already embarked on the process of digital transformation prior to the COVID-19 pandemic, but the transformation was proceeding slowly resulting in most AHP services being unprepared for telehealth implementation when the pandemic hit. While healthcare systems typically need to develop policies and workforce training to move to a new model of care delivery [5], some of these phases may have been bypassed during the COVID-19 pandemic because of the urgent change required. Therefore, AHP services may have faced challenges of organisational readiness, such as ambiguity related to patient selection and suitability, technological resources necessary to implement telehealth, lack of workforce training and telehealth-specific guidelines [6]. For example, not all AHPs were provided with telehealth guidelines from their professional bodies. Those AHP groups for which guidelines were available may have not found adequate support in them as guidelines were found to lack information on some topics and had areas of ambiguity [7]. In addition, with a lack of telehealth training within the AHP curricula [8], many AHPs, may have not been trained to deliver telehealth and learned how to undertake telehealth appointments on-the-job [9]. Taken together, these may have constituted barriers to the acceptance of telehealth into the mainstream healthcare system limiting its implementation on a much greater scale.

The potential unpreparedness of the AHP services may have had other consequences. As a result of lack of guidelines and training, AHPs may have struggled with translating their hands-on skills to digital environments [10] and, consequently, may have not been fully equipped to fulfill their ethical obligation of non-maleficence thereby exposing patients to the risk of harm. Due to the lack of prior telehealth-related experience, AHPs may have experienced low self-efficacy in providing telehealth consultations [11] thereby undermining the quality and safety of services leading to poorer patient outcomes. Ambiguity surrounding the identification of patient groups potentially suitable for telehealth may have caused patients with certain conditions, disabilities, and poor digital literacy to be left behind in the migration to telehealth. This potential inequity of access to remote healthcare may have widened health inequalities [12]. While telehealth may result in reduced barriers to healthcare for some it also has the potential to increase barriers for certain populations, such as older people [13] and people with disabilities [14].

Digital healthcare technologies have been identified by the NHS as a “new means of addressing the big healthcare challenges of the twenty-first century” [15] with programmes available to increase digital readiness across the health and social care workforce [16]. Digital transformation was cited as one of the priorities of the 2019 NHS Long Term Plan, with face-to-face outpatient appointments set to reduce by a third by 2024 and most patients expected to receive a “digital first” option by 2029 [17]. Specifically related to AHPs, there have been recent NHS publications, the AHPs strategy [18] and the digital framework for AHPs [19], which have focused on utilisation of digital technology.

However, NHS organisations are likely to face significant challenges in working toward digital expansion [20]. For example, digital transformation requires clear and consistent guidelines, which are not yet in place. With the current lack of a plan by the professional bodies to improve AHPs’ digital skills [20], there is a need to prepare a strategic workforce plan to support digital transformation. This is essential for the success of the NHS digital transformation as previous digital strategies have been unsuccessful, because they predominantly focused on technology neglecting the adaptive clinical changes [20, 21]. There have been concerns that the digital transformation will widen the digital divide for certain populations when telehealth becomes ubiquitous [22]. Understanding the possible compensation measures for different populations is essential to address possible health inequalities [12].

Thus, it is important to understand the organisational preparedness of AHP services in the use of telehealth as this information will serve as a basis for the development of guidelines and training programs required for a sustainable, long-term telehealth implementation. The aim of this study was to explore the UK AHP services’ organisational telehealth readiness, focusing on guidelines implementation and AHP staff training, through the perspectives of NHS AHPs and AHP service managers.

## Methods

### Survey design and recruitment

This cross-sectional study was conducted among the 14 professions considered AHPs within NHS England [23] and AHP service managers. Participants having both a clinical and management role were asked to choose whether to answer the survey as a clinician or as a service manager. Managers in charge of multiple services were asked to provide responses for up to three services. Ethical approval was granted by the Staffordshire University Research Ethics Committee (Reference: SU20-153-RN).

The online survey was created using Qualtrics (Qualtrics International, USA) and was launched online on the 7th of May 2021 and remained open for 8 weeks. The survey was distributed via all 14 UK AHP professional bodies, the AHP federation, England NHS lead networks, the AHP Public Health England Lead networks, The Orthotic and Prosthetic Networks, The National Orthotics Managers’ Association Group, Sharing Thoughts for Optimising Recovery and Engagement (RESTORE) network, NHS AHP collaboration platform and the Physiotherapy Research Society. All NHS trusts (an organisational unit within the NHS) in the UK were contacted and requested to distribute the survey within their AHP workforce. Informed consent was obtained from all participants prior to completing the survey.

### Survey development

The survey covered questions related to telehealth implementation, financial and technical considerations and was developed based on the findings of our previous scoping review [7]. The survey, consisting of a combination of multiple-choice questions, multiple answer questions and additional free text fields, was subdivided into seven sections: (1) prevalence of telehealth consultations in AHP services, (2) barriers to the use of telehealth consultations for patients and clinicians/AHP services, (3) perceived benefits and disadvantages to the use of telehealth consultations for patients and clinicians/AHP services, (4) available telehealth guidelines for clinicians, (5) telehealth consultation training for clinicians, (6) funding for telehealth consultations, and (7) effect of telehealth consultations on healthy behaviour conversations. This paper will focus on survey sections 4 and 5, which are available in Additional file 1.

### Data analysis

Data were analysed using SPSS Statistics, version 27.0. Descriptive statistics were calculated for the demographic characteristics of the entire sample (e.g., NHS role, UK region, clinical background). After classifying participants based on their role (clinician or manager), descriptive statistics were computed for the two groups. Contingency table analyses were conducted to explore any differences in terms of guidelines implementation between AHP professions and different types of NHS services. All variables were presented as frequencies and corresponding percentages.

## Results

### Participants

658 participants answered the survey, 539 (82%) answered as clinicians and 119 (18%) as managers, representing 168 AHP services. Most respondents were working as a physiotherapist, speech and language therapist or occupational therapist. Similarly, most managers oversaw physiotherapy, occupational therapy and speech and language therapy services. Most respondents worked in acute/hospital outpatient, community and acute/hospital inpatient NHS settings, with some participants working across multiple settings. Participants were working across the different UK nations and regions, predominately in England and in four of its regions (south–east, south–west, north–west and Yorkshire and Humber). Most respondents were senior clinicians (e.g., UK pay bands 6, 7 and 8a) and their experience ranged from 1 to 25 years. The demographic characteristics of the survey participants are summarised in Table 1.

**Table 1:** Demographic characteristics of survey participants

Characteristics	*n* (%)	Characteristics	*n* (%)
**NHS role**		**Pay banding**	
Clinicians	539 (82%)	Band 7	238 (36.2%)
Managers	119 (18%)	Band 6	200 (30.4%)
		Band 8a	99 (15.0%)
**UK region**		Band 5	56 (8.5%)
England—South–East	104 (15.8%)	Band 8b	29 (4.4%)
England—South–West	82 (12.5%)	Band 8c	17 (2.6%)
England—North–West	95 (14.4%)	Prefer not to say	10 (1.5%)
England—Yorkshire and Humber	93 (14.1%)	Band 8d	6 (0.9%)
England—East Midlands	73 (11.1%)	Band 9 or above	3 (0.5%)
England—West Midlands	68 (10.3%)		
England—London	61 (9.3%)	**Years of service**	
England—East	31 (4.7%)	1–5 years	117 (17.8%)
Scotland	23 (3.5%)	16–20 years	106 (16.1%)
England—North–East	13 (2.0%)	6–10 years	106 (16.1%)
Wales	9 (1.4%)	21–25 years	92 (14.0%)
Northern Ireland	6 (0.9%)	11–15 years	82 (12.5%)
		31 + years	67 (10.2%)
**Clinical background**		26–30 years	67 (10.2%)
Physiotherapist	222 (33.7%)	< 1 year	21 (3.2%)
Speech and Language therapist	108 (16.4%)		
Occupational therapist	102 (15.5%)	**Type of NHS service**	
Dietitian	71 (10.8%)	Physiotherapy	48 (28.6%)
Prosthetist/Orthotist	56 (8.5%)	Occupational therapy	29 (17.3%)
Chiropodist/Podiatrist	39 (5.9%)	Speech and language therapy	23 (13.7%)
Orthoptist	38 (5.8%)	Prosthetics/Orthotics	18 (10.7%)
Radiographer	12 (1.8%)	Chiropody/Podiatry	15 (8.9%)
Paramedic	3 (0.5%)	Dietetics	13 (7.7%)
Drama therapist	2 (0.3%)	Other*	8 (4.8%)
Osteopath	2 (0.3%)	Orthoptics	7 (4.2%)
Art therapist	1 (0.2%)	Radiography	6 (3.6%)
Music therapist	1 (0.2%)	Music therapy	1 (0.6%)
Operating department practitioner	1 (0.2%)		
**NHS setting**			
Acute/hospital outpatient	315 (50.1%)		
Community	300 (47.7%)		
Acute/hospital inpatient	192 (30.5%)		
Primary care	69 (11.0%)		
Domiciliary	50 (7.9%)		
Social care	10 (1.6%)		
Education	1 (0.2%)		
Research and L&D	1 (0.2%)		

^*^Community pain service, diabetic foot service, hand therapy, integrated care and assessment treatment service (ICATS), stroke service, first contact practitioner (FCP) service, radiotherapy and optometry (0.6% each)

### Telehealth guidelines implementation

Of the 539 clinicians who took part in the study, 87.4% (*n* = 471) reported using telehealth for patient consultations. The majority of those clinicians using telehealth (64%, *n* = 308) reported that their services had implemented telehealth guidelines. The remaining proportion of clinicians reported either that their services had not implemented telehealth guidelines (21%, *n* = 98) or that they did not know whether telehealth guidelines had been implemented (15%, *n* = 73). Similarly, most managers reported that their services had implemented telehealth guidelines (82%, *n* = 96), 15% (*n* = 17) answered that their services had not implemented guidelines and 3% (*n* = 4) were not sure. The highest implementation rate of guidelines was observed in speech and language therapy (clinicians, 84%; managers 89%) occupational therapy (clinicians, 60%; managers: 70%), physiotherapy (clinicians: 66%; managers: 81%) and chiropody/podiatry (clinicians, 67%; managers, 86%) services. The highest non-implementation rate was recorded in orthoptic (43%) and radiography (75%) services among clinicians and in dietetic services (21%) among managers.

### Telehealth guidelines production

Most of those clinicians and managers whose service had implemented telehealth guidelines were using guidelines produced by their NHS trust (clinicians, 70%, *n* = 212; managers, 64%, *n* = 60), followed by their service team (managers, 51%, *n* = 48), the AHP service manager (clinicians, 29%, *n* = 88; managers, 22%, *n* = 21), and the AHP professional body (clinicians, 28%, *n* = 86; managers, 31%, *n* = 29).

### Telehealth guidelines use

**Fig. 1:**
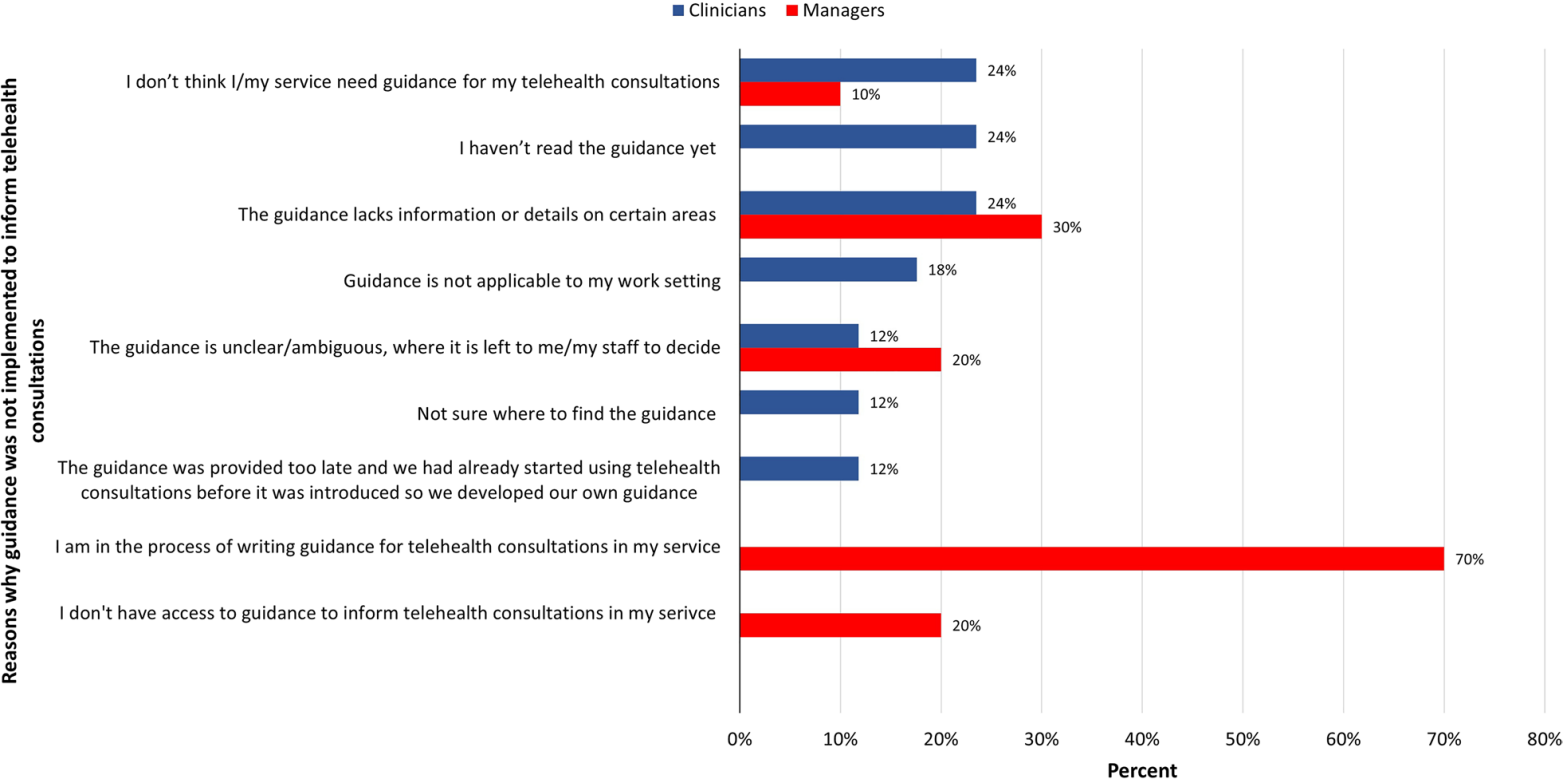
Reasons why clinicians and service managers did not implement guidance to inform telehealth consultations

Nearly all clinicians whose service had implemented guidelines used them in their own consultations. Among those who did not, the most common justifications were that they thought they did not need guidelines to deliver telehealth consultations, they had not read the guidelines yet and that guidelines lack information on certain aspects (Fig. 1). Managers had not implemented guidelines in their service, mainly because they were still in the process of writing them (Fig. 1).

### Presence of areas of ambiguity or lack of information in current telehealth guidelines

**Fig. 2:**
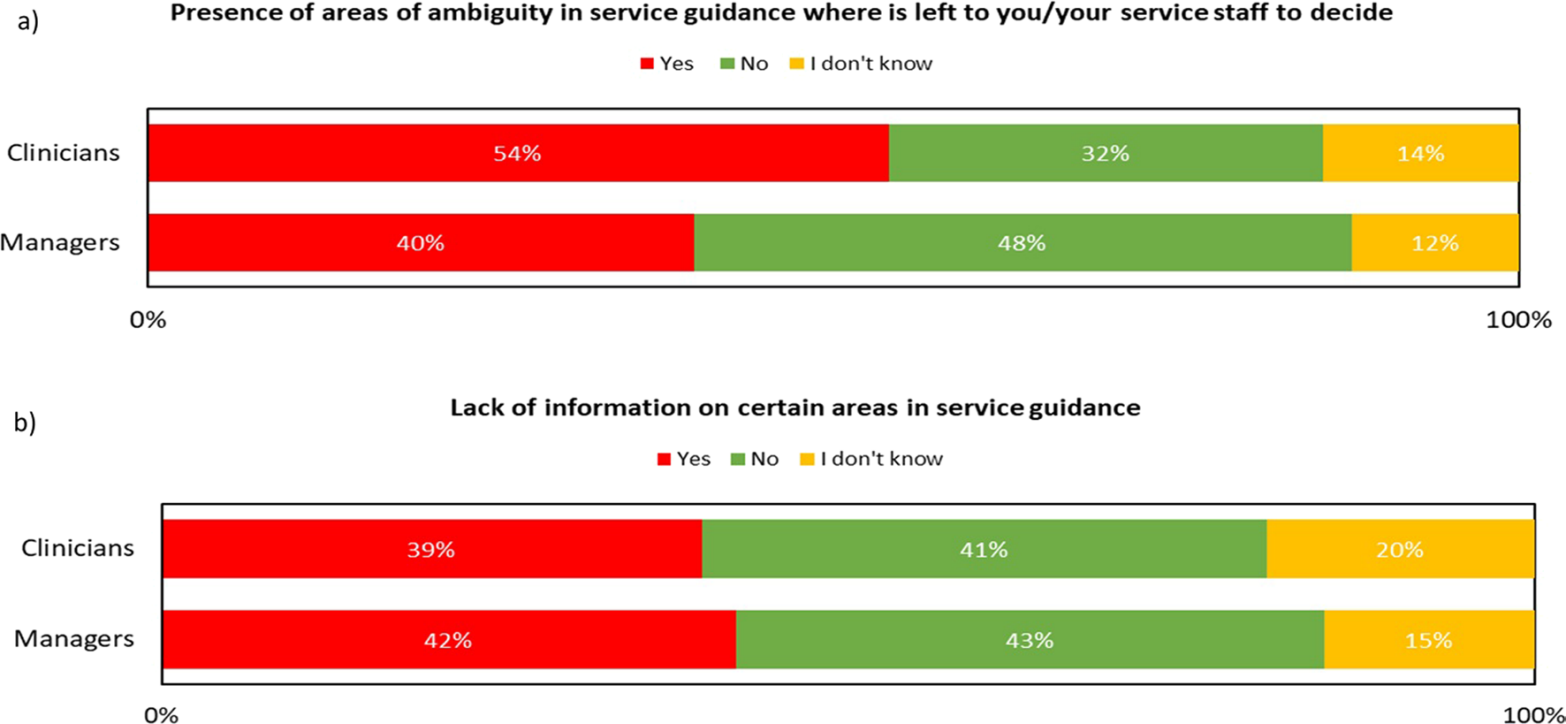
**a** Presence of ambiguity in service guidance according to clinicians and managers; **b** Lack of information on certain areas in service guidance according to clinicians and managers

More than half of the clinicians reported that guidelines have ambiguous areas, whereas the majority of the managers reported the opposite opinion (Fig. 2a). There was uncertainty among clinicians and managers as to whether there was a lack of information within the guidelines (Fig. 2b).

### Areas of ambiguity and lacking information in the telehealth guidelines

**Fig. 3:**
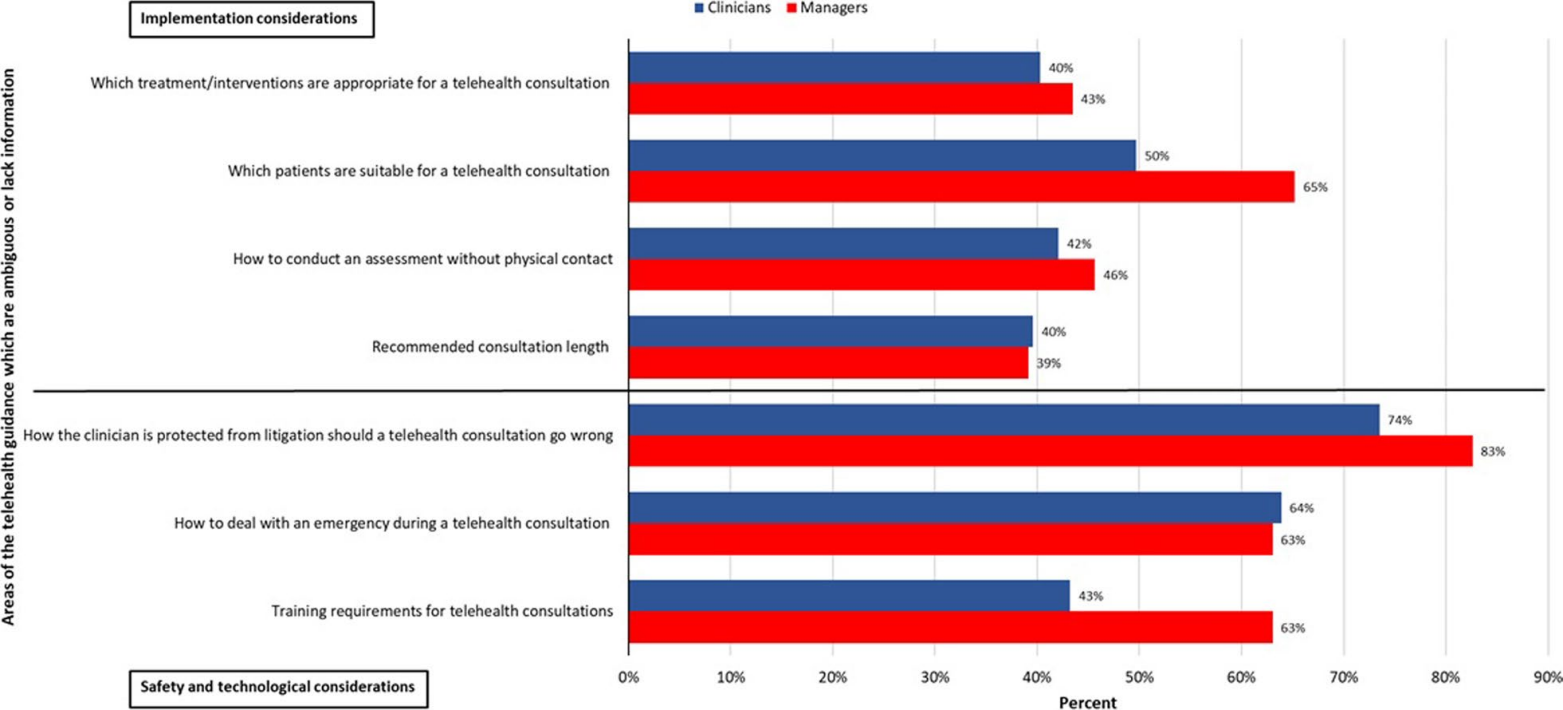
Areas of telehealth guidance which are ambiguous or lack information according to clinicians and managers

The largest percentage of both clinicians and managers reported that there is ambiguity or lack of information in the current guidelines on how clinicians are protected from litigation (Fig. 3). Other areas most frequently reported by both groups as ambiguous or lacking information were how to deal with an emergency, which patients are suitable for telehealth and telehealth training requirements (Fig. 3).

### Telehealth aspects on which current guidelines provide advice

**Fig. 4:**
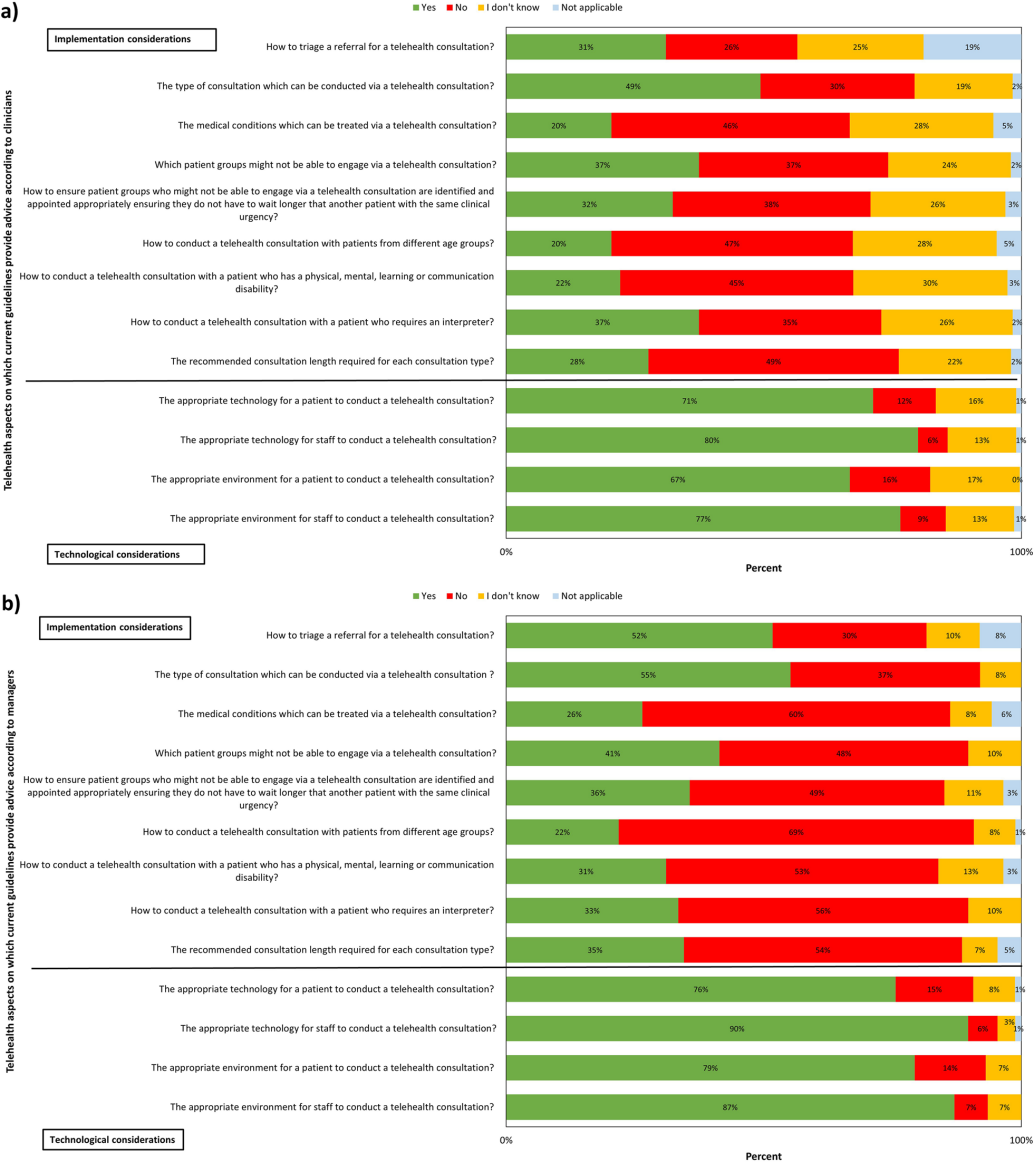
**a** Telehealth aspects on which telehealth guidance provides advice according to clinicians; **b** Telehealth aspects on which current telehealth guidance provides advice according to managers

According to both clinicians and managers (Fig. 4a, b), the telehealth aspects on which current guidelines most frequently provided advice were the appropriate technology and environment to conduct a telehealth consultation for staff and patients. The least frequently reported aspects for both groups were the recommended consultation length, how to conduct telehealth consultations with patients from different age groups, or with patients having disabilities or requiring an interpreter, the medical conditions that can be treated via telehealth, which patient groups may not engage with telehealth, and how to ensure that individuals from these groups are identified and appointed appropriately. Furthermore, approximately 30% of clinicians reported that they did not know whether guidelines provided advice on how to conduct a remote consultation via telehealth with patients with disabilities or from different age groups and the medical conditions that can be treated through telehealth.

### Telehealth consultations for people with disabilities or requiring interpreter support

Many clinicians reported that they did not know (33%, *n* = 94) whether the guidelines advise on how patients with physical, mental, learning or communication disabilities or those requiring an interpreter should be identified and triaged. Other common responses among clinicians were that patients are offered a telehealth consultation first and if this is unsuccessful, they are then offered a face-to-face consultation (28%, *n* = 82) and that guidelines do not offer advice on which consultation method these patients should receive (26%, *n* = 75). The most common responses among managers were that guidelines do not specify the consultation type these patients should receive (36%, *n* = 31) and that these patients are usually offered telehealth consultations, but face-to-face consultations are then offered if the first modality is unsuccessful (30%, *n* = 26).

### Training on telehealth aspects

**Fig. 5:**
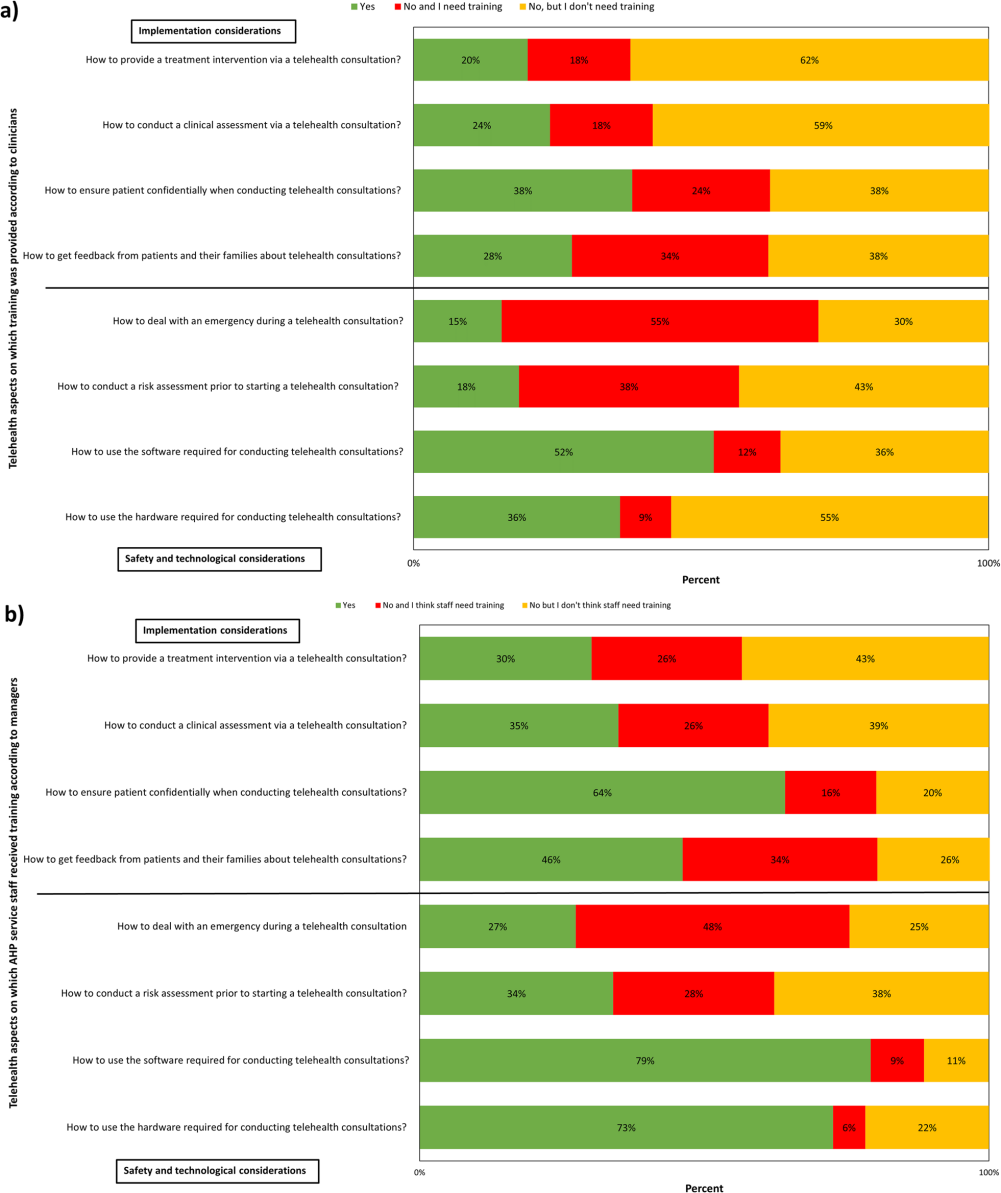
**a** Telehealth aspects on which clinicians received training; **b** Telehealth aspects on which AHP service staff received training according to managers

Most clinicians reported that they had not received training in all telehealth aspects listed (Fig. 5a). Clinicians considered training on some aspects as necessary (e.g., how to deal with an emergency during a telehealth consultation, how to conduct a risk assessment before a telehealth consultation and how to get feedback from patients and their families). However, training was not considered necessary by clinicians in most telehealth aspects, such as how to provide a treatment intervention or conduct an assessment via telehealth and how to use the hardware required for telehealth consultations. The telehealth aspect in which the highest proportion of clinicians had received training was how to use the software required for telehealth.

According to managers, their AHP service staff had received training on some telehealth aspects, such as how to use both the software and hardware required for telehealth consultations, how to ensure patient confidentiality during telehealth consultations, and how to get feedback from patients and their families (Fig. 5b). Most managers reported that AHPs had not been trained in the other telehealth aspects. However, while training on some of these aspects was considered as not necessary (e.g., how to provide a treatment intervention or conduct an assessment and how to conduct a risk assessment prior to a face-to-face consultation), most managers reported that AHPs would require training on how to deal with an emergency during a telehealth consultation.

## Discussion

In response to the COVID-19 pandemic, UK NHS AHP services were tasked with rapidly implementing telehealth patient consultations. This may have caused usual implementation strategies for practice change to be bypassed. Despite these challenges, 64% and 82% of the AHP services who responded to this survey have implemented telehealth guidelines according to NHS clinicians and managers, respectively. AHPs showed a positive attitude toward telehealth guidelines with the majority using them for their own consultations. Although this may indicate a good overall level of preparedness and acceptance toward telehealth across NHS AHP services, there were differences in terms of guidelines implementation between services from different AHP groups. The highest non-implementation rates were observed in the smaller AHP professions’ services, such as orthoptic, radiography and dietetic services. This may be the result of inequalities between large and small AHP services in terms of financial and personnel resources for the development of guidelines [7]. In addition, some of these findings are based on a small number of respondents, and therefore, future studies should confirm the implementation rates in these small professions.

Our findings are in line with previous international studies that identified lack of awareness and lack of familiarity with the guidelines as barriers to guidelines implementations by healthcare professionals [24]. The latter reason may also indicate that clinicians shifted from in-person care to telehealth without having had the time or the opportunity to acquire new skills and knowledge necessary before embarking on a different modality of service provision. This hypothesis seems to be supported by the finding that the reason given by most managers for not having implemented telehealth guidelines in their service was that they were still in the progress of writing them. Taken together, these results indicate that NHS AHP services introduced telehealth with very little preparedness and readiness, which resulted in them being inadequately equipped to provide telehealth. Therefore, it is essential for AHP services to invest in organisational and infrastructural resources to ensure that telehealth can be successfully embedded into long-term service delivery.

Most clinicians and service managers reported that the guidelines they were using had been developed by their NHS trust, the service staff or the AHP service manager. With telehealth guidelines varying from one NHS trust to another or within the same trust from one service to another, every AHP service appears to work in isolation and there may be a lack of commonly accepted telehealth standards across the NHS AHP services. This may cause health inequities between large-urban and small, rural and potentially underfunded NHS trusts or services. To address existing disparities or misalignments, there is a need for the NHS and AHP professional bodies to cooperate in the development of common guidelines.

Although clinicians and managers appeared to have contrary opinions about the presence of ambiguous areas in the guidelines, there appears to be an agreement between them in respect to the areas lacking information and providing ambiguous advice. These mainly pertained to telehealth safety considerations, training requirements and patient eligibility criteria. This reflects the findings of our previous review including international guidelines [7] and indicates that future guidelines should include more attention to these aspects which require further clarification.

There was an agreement between clinicians and managers on the telehealth aspects included in the current guidelines, suggesting that current guidelines have adopted a one-size-fits-all approach to telehealth consultations, neglecting the diverse needs of different clinical groups and concentrated on hardware and software [21]. People with a wide range of disabilities (e.g., physical, mental, learning or communication disorders) are currently excluded from telehealth guidelines. Most clinicians and managers reported that current guidelines do not provide advice, or that they did not know if they provided advice, on how these population groups should be identified and triaged. Should a person with a disability or poor language proficiency experience accessibility or communication barriers, this is likely to result in delays in receiving care which may lead to adverse outcomes. It is vitally important that barriers to using digital technologies are addressed in order not to leave behind 14.1 million UK people (21%) with disabilities in the transition to telehealth [25]. In addition, it should be noted that most previous digital transformation projects failed, because they did not redesign clinical processes when disruptive technology was implemented [20, 21]. As this could significantly undermine the success of the NHS Long Term Plan [17], it is crucial that NHS organisations adopt digital inclusion approaches, such as digital technologies more accessible for people with disabilities, digital skills training, and “digital carers” to help patients access health services [12, 26].

In our survey, responses from both clinicians and managers highlighted that AHPs had not received training in most telehealth aspects and indicated that prior training mostly focused on technology-related aspects. Both clinicians and managers reported that more training would be required in safety-related aspects of telehealth consultations (e.g., how to deal with an emergency and how to conduct a risk assessment during a consultation). Appropriate training for telehealth consultations is considered essential [27] and our findings are in line with previous research from the United States in which healthcare professionals reported needing skills to manage emergencies during telehealth [28]. These results indicated that the skills and knowledge needed to respond to emergencies in a virtual environment were different from those used during face-to-face consultations [28]. This suggests that AHP undergraduate curricula and further education training should offer not only hands-skills sessions but should include telehealth education and training to allow future AHPs to safely and confidently practice in an increasingly digital world. In addition, NHS organisations should offer their current staff appropriate emergency response training, which would equip them to deal with emergencies and improve patient safety during telehealth consultations.

This study was the first to explore the organisational readiness of the UK NHS AHP services in terms of guidelines implementation and workforce training through the perspectives of NHS AHPs. Although this survey was widely distributed, the survey respondents represent only a small proportion of the AHPs employed in the NHS and predominantly worked in England’s services. Therefore, caution is required to generalise the results of this study to other UK nations. The online nature of the survey may have introduced a bias toward those AHPs who are more comfortable with communication technologies and telehealth guidelines. Similarly, personal beliefs and attitude of the healthcare professional respondents toward telehealth and their organisations’ baseline readiness may have influence the results of this study. In addition, the majority of the responses were from the physiotherapy, occupational therapy and speech and language professions. While this was not unexpected, as these are professions with the largest workforces among the 14 UK NHS AHPs and the ones which utilise telehealth widely, the results are, therefore, more reflective of the perspectives of these professions than of all the 14 professions.

Despite the good level of guidelines implementation and the positive trends among AHPs toward the use of guidelines, the findings of this study indicate that NHS AHP services and their staff are not yet fully equipped with clear and comprehensive guidelines and with the skills necessary to deliver telehealth consultations. Most guidelines are issued at a local level, highlighting the necessity of central uniform AHP guidelines. There is limited readiness of the clinical processes, which manifested as poor understanding of patients’ eligibility criteria and failure to tailor telehealth consultations to vulnerable population groups. Consequently, there is a danger that digital expansion will widen health inequalities. Inclusive innovation strategies are needed within the NHS to successfully deliver the digital transformation of AHP services.

## Supplementary Information

**Additional file 1.** Survey questions.

## Data Availability

The data for this paper were obtained via a survey and it is currently being analysed. Although the authors are not providing the data publicly, they will be happy to share the data outlined within this paper on request.
