# Supplementary figures and images for: Do UK Allied Health Professionals (AHPs) have sufficient guidelines and training to provide telehealth patient consultations?
Source: Hum Resour Health. 2022 Dec 5;20:82. doi: 10.1186/s12960-022-00778-1 (PMC9721053; doi:10.1186/s12960-022-00778-1)

**Additional file 1. Survey**

**
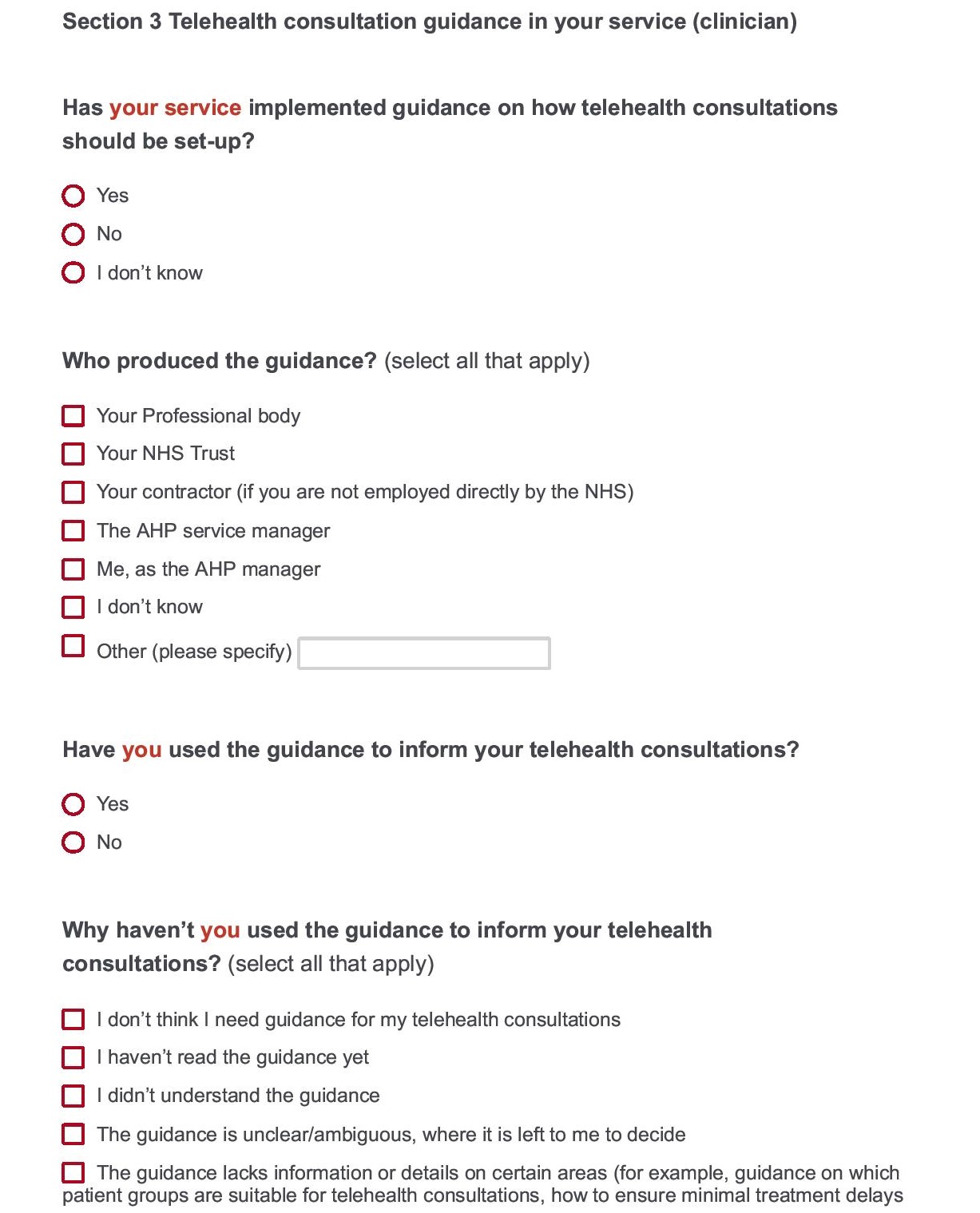
**


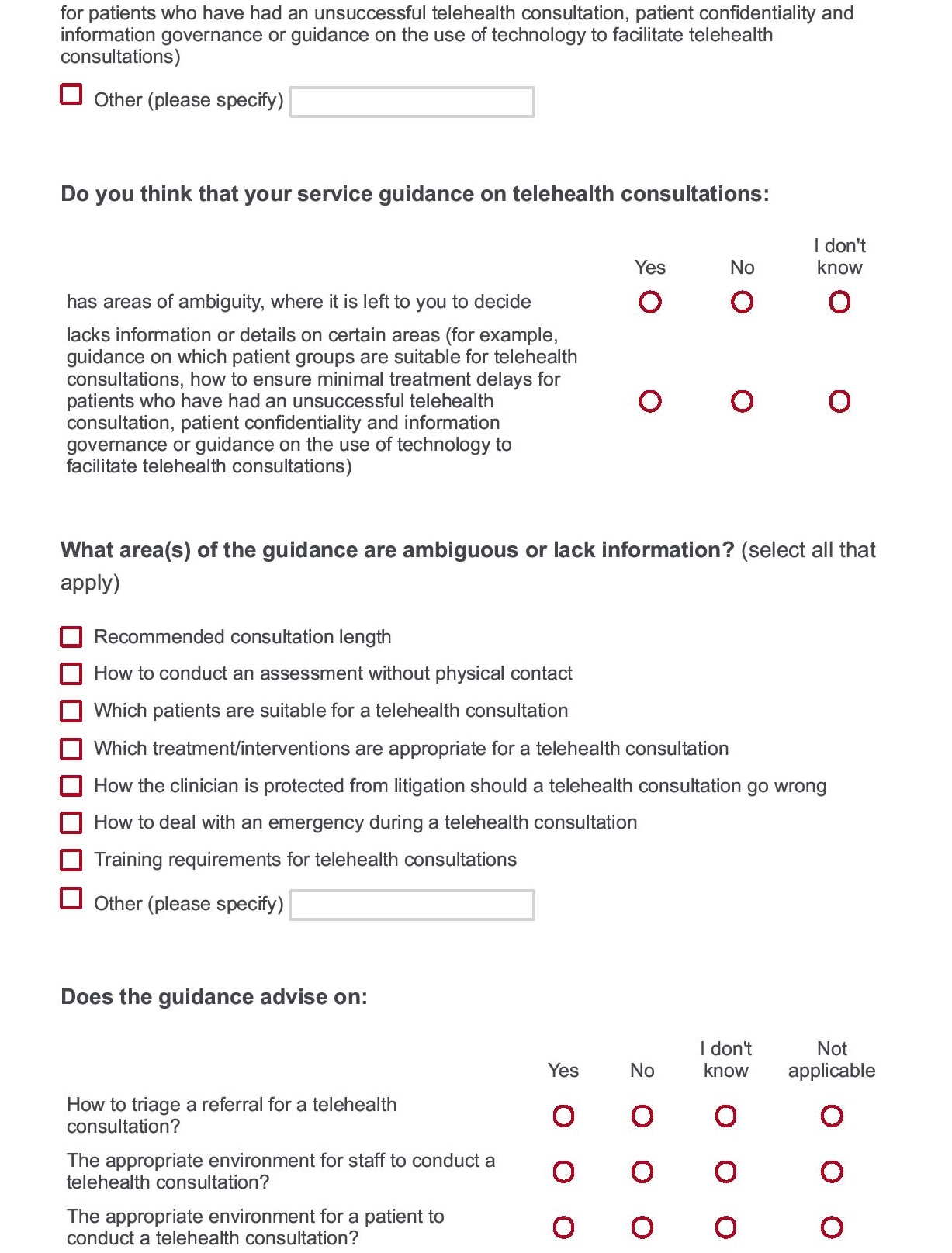


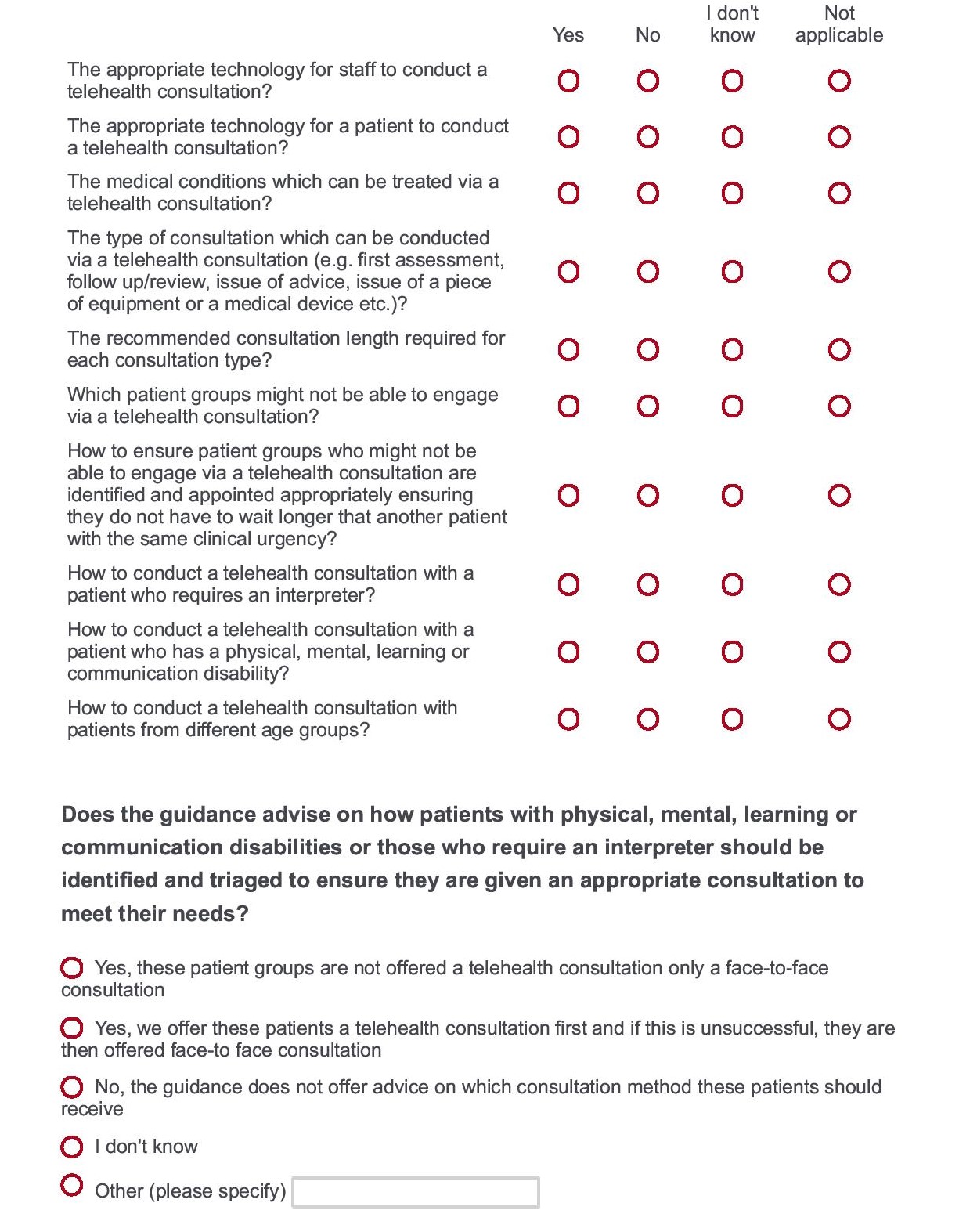


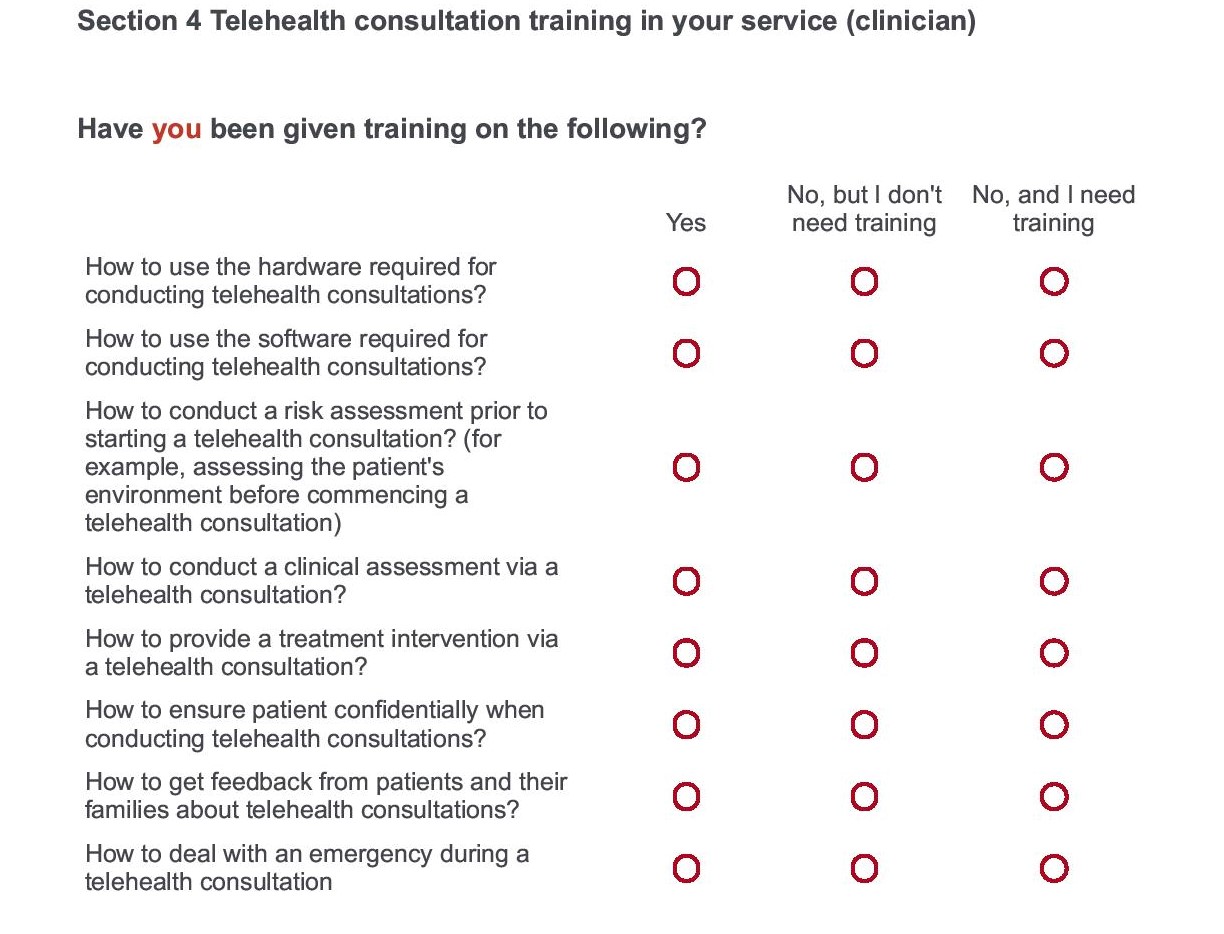

Supplement: Supplementary file 1 — Additional file 1. Survey questions. [file 12960_2022_778_MOESM1_ESM.docx]
